# Inoculation of Tuberculosis of the Gallinæ in the Mammifers2Ibid.

**Published:** 1896-05

**Authors:** 


INOCULATION OF TUBERCULOSIS OF THE GALLINÆ IN THE MAMMIFERS.[2] Cadiot, Gilbert, and Roger (*Société de Biologie*, meeting of February 8, 1896) state that the study of aviary tuberculosis has led them to the opinion that this bacillus and that of tuberculosis of the mammifers are two varieties or two kinds of the same species. They admit that they are from the same origin, but that their transformation is possible. The authors sustain their opinion by a collection of eighty-five cases. Everybody can see thereby that tuberculosis of the gallinæ is easily inoculated in a rabbit, but not so readily in a guinea-pig. After different transitions in the mammifers it may become very active in this rodent, and create in him, like the human virus, the development of visceral granulation. At the same time it may lose its noxious actions in the gallinæ. To observe the same modification in the virulency of the aviary bacillus, it will be necessary to continue observations for years, and to recollect that tuberculosis of the gallinæ must not be considered as a definite virus, always resembling itself; it varies remarkably between one birth and another. Reciprocally the mammifers of the same species are far from having the same disposition or the same resistance to a given virus. Consequently, we must consider that the individual susceptibility is of great influence.

